# Supplementary material for: Qualities and Inequalities in Online Social Networks through the Lens of the Generalized Friendship Paradox
Source: PLoS One. 2016 Feb 10;11(2):e0143633. doi: 10.1371/journal.pone.0143633 (PMC4749216; doi:10.1371/journal.pone.0143633)
Supplement: S1 File — (DOCX) [file pone.0143633.s001.docx]

Supporting Information File

Instructions for Accessing the Data

The data reported in the paper by Kwak et al. can be obtained using the Twitter API in the following manner. The dataset contains two parts that we used in this work: tweet data and social network data.

To obtain the tweet data, the Twitter API can be used to obtain the top 10 trending topics for every 5-minute interval between July 6, 2009 and July 31, 2009, and then the query string for each trending topic title can be used to search for relevant tweets in said interval. Tweets are deemed to be spam and are not included in the dataset if either the user sending the tweet has been on Twitter for less than one day or if the tweet contains three or more trending topics.

The bulk of the social network data (follower/followee relationships) could be obtained using the Twitter API to obtain all user profiles and follower relationships created before June 31, 2009 by performing a crawl (breadth-first search) starting from Perez Hilton. Additional profiles are gathered for users that sent a tweet with a trending topic and that do not appear in the giant connected component (i.e., users that are not reached starting from Perez Hilton).
